# Efficacy and safety of esketamine for pain control after cesarean delivery: a systematic review and meta-analysis of randomized controlled trials

**DOI:** 10.3389/fphar.2025.1708221

**Published:** 2025-12-01

**Authors:** Tingyu Jiang, Rui Zhao

**Affiliations:** 1 Department of Anesthesiology, Guangzhou Panyu District Maternal and Child Health Hospital, Guangzhou, China; 2 Department of Anesthesiology, Second Affiliated Hospital of Guangzhou Medical University, Guangzhou, China

**Keywords:** esketamine, cesarean delivery, pain, depression, meta-analysis

## Abstract

**Objective:**

This study aimed to investigate the efficacy and safety of esketamine in reducing postpartum pain and depression in patients undergoing cesarean delivery (CD).

**Methods:**

Web of Science, Embase, PubMed, and Cochrane Library were searched from the inception of each database up to September 2025 to select relevant studies. Randomized controlled trials (RCTs) that assessed the efficacy of esketamine for pain control in patients undergoing CD were selected. The outcomes included scores of postpartum pain, the incidence of depression after surgery, scores on the Edinburgh Postnatal Depression Scale, and the incidence of adverse events before and after surgery. The stability of the results and potential sources of heterogeneity were investigated by subgroup and sensitivity analyses.

**Results:**

This meta-analysis included ten studies comprising 15 intervention groups. A total of 2218 individuals undergoing CD were involved. Compared with the control group, esketamine showed relatively favorable effects on controlling rest pain at 24 h (SMD = −0.42; 95% CI: −0.69 to −0.16; P < 0.00001; I^2^ = 84%), movement-evoked pain (MEP) at 24 h (SMD = −0.68; 95% CI: −1.29 to −0.07; P < 0.00001; I^2^ = 92%), rest pain at 48 h (SMD = −0.22; 95% CI: −0.38 to −0.06; P = 0.006; I^2^ = 54%), and MEP at 48 h (SMD = −0.63; 95% CI: −1.07 to −0.20; P < 0.00001; I^2^ = 85%). In contrast to the control group, EPDS scores in the esketamine group were lower (SMD = −0.21; 95% CI: −0.39 to −0.04; P = 0.02; I^2^ = 45%). Subgroup analyses indicated that pregnant women aged over 30 years who received esketamine had a higher incidence of dizziness (RR = 2.08; 95% CI: 1.26 to 3.34; P = 0.004; I^2^ = 64%). The quality of evidence was moderate for six outcomes, and low for three outcomes according to the GRADE assessment.

**Conclusion:**

As a therapy for postoperative analgesia, esketamine shows relatively favorable efficacy in pain control and is associated with a lower risk of postpartum depression. However, this result is unstable. Furthermore, esketamine may increase the risk of dizziness in pregnant women aged over 30 years. Since all the experiments in this study are conducted in China, there are certain regional limitations.

## 1 Background

Cesarean delivery (CD) is one of the most painful surgeries. With advances in medical science and technology, anesthesiologists are increasingly focusing on optimizing pain management and minimizing anesthesia-related complications in parturients. Due to physiological differences, women tend to be more sensitive to pain than men (Athnaiel et al., 2023). Neuraxial anesthesia is commonly used for CD. However, because neuraxial anesthesia acts slowly and does not fully block visceral nerves, it often causes pain and nausea in parturients during fetal delivery and the cleansing of the abdominal cavity. Furthermore, due to its clinical characteristics, CD is more likely to cause significant hemodynamic fluctuations and postoperative nausea and vomiting (PONV) compared to other abdominal surgeries. Thus, CD can severely impact both postoperative recovery and the psychology of parturients. In addition, the intense pain caused by CD may trigger a strong stress response in parturients, thereby leading to various pathological changes and even increasing the risk of postpartum depression in these patients (Joshi et al., 2025; Miller et al., 2022). Therefore, effective pain management is critical for the postoperative recovery of patients undergoing CD. Esketamine, a cyclohexanone derivative, is effective in controlling pain, relieving anxiety, inhibiting sympathetic nerves, and stabilizing haemodynamics. In recent years, esketamine has been increasingly used for anesthesia in CD, and it has diverse routes of administration, including intravenous and neuraxial anesthesia. Moreover, it has also been used in postoperative patient-controlled analgesia (PCA). It has been demonstrated that esketamine can alleviate pain and depression in patients following CD, and lower the Edinburgh Postnatal Depression Scale (EPDS) scores without increasing the incidence of adverse effects such as PONV (Yang et al., 2024). However, another study reports that esketamine does not notably reduce postoperative pain (Liu et al., 2023). Moreover, no statistical difference in pain scores between postoperative days 1 and 2 is found in these studies, and only the efficacy of esketamine in reducing depression is observed. Because this drug has both analgesic and antidepressant effects, and pain and depression are somewhat correlated, most of the results regarding its antidepressant effects are exploratory (Mion and Himmelseher, 2024). In the study by Shen et al., for example, the drug only shows analgesic effects and does not exhibit antidepressant effects (Shen et al., 2022).Wang et al. (Wang et al., 2021) have published a meta-analysis including 12 studies. Their study demonstrates that esketamine is effective in reducing opioid consumption after surgery. However, other meta-analyses have revealed that the analgesic efficacy of esketamine is not significant. Due to the variations in dosages, as well as the routes and time of administration, the current evidence regarding the analgesic efficacy of esketamine remains inconclusive. Thus, this study aimed to assess the analgesic efficacy and safety of esketamine and investigate the optimal route of administration of esketamine.

## 2 Methods

### 2.1 Protocol and registration

The study was registered in the International Prospective Register of Systematic Reviews (No. CRD42024620674). This study was reported according to the Preferred Reporting Items for Systematic Reviews and Meta-Analyses (PRISMA) guidelines, its protocol, and the PRISMA extension for meta-analyses (Page, 2020).

### 2.2 Search strategy

Randomized controlled trials (RCTs) that assessed the efficacy of esketamine for pain control in patients undergoing CD were searched from Embase, Web of Science, Cochrane Library, and PubMed from their inception to September 2025. The keywords included esketamine, kataved, cesarean delivery, S-Ketamine, abdominal delivery, pain, and ache. No restrictions on language or region were applied. Moreover, non-English studies should provide an adequate English abstract. Additionally, reference lists of the selected RCTs were manually searched to identify any additional eligible studies. The search strategy is illustrated in Supplementary Table S1.

### 2.3 Study selection

After reviewing titles, abstracts, and full texts, eligible RCTs were included according to the inclusion and exclusion criteria.

The inclusion criteria were as follows: (i) participants: women aged 18 years or older undergoing CD; (ii) intervention and comparison: the intervention group received esketamine either intraoperatively or postoperatively, while the control group received placebo or non-esketamine analgesics; (iii) studies reported one of the following outcomes: postoperative pain scores measured by VAS or NRS (the primary outcomes); the incidence of adverse events, intraoperative blood loss, operation duration, and EPDS scores (the secondary outcomes); (iv) study design: RCTs.

The following studies were excluded: (i) non-RCTs, retrospective studies, animal studies, and reviews; (ii) studies that involved patients who were unable to participate in the trial for such reasons as mental illness; (iii) studies with unavailable data from the original authors, and with inaccurate or incomplete outcome measurements; (iv) duplicate publications.

### 2.4 Data extraction

Two reviewers (Jiancheng Tang and Tingyu Jiang) independently extracted data using a standardized Excel template. The extracted data were as follows: (i) publication details: first author, title, and publication year; (ii) study characteristics: study design, duration of the intervention; (iii) characteristics of patients: presence or absence of hyperthyroidism, hypertension, scarred uterus, as well as number of participants and age; (iv) interventions: route of administration, frequency, drug name, and dosage for the control groups; treatment regimen, frequency of administration, and dosage for the intervention group; (v) primary and secondary outcomes: continuous data that were presented as means or standard deviations, and categorical data that were presented as event counts and total sample sizes. Any disagreements were addressed by a third reviewer.

### 2.5 Quality assessment

The quality of the included RCTs was assessed independently by two reviewers (Tingyu Jiang and Rui Zhao) using the Cochrane Risk of Bias tool (Cumpston et al., 2022). Moreover, the evaluation results were cross-checked by the two reviewers. The quality assessment contained seven domains: blinding of participants and personnel, random sequence generation, blinding of outcome assessment, reporting bias, incomplete outcome data, allocation concealment, and other bias. The risk of bias in the included studies was classified as low, high, or unclear. Any disagreements were addressed by a third reviewer.

### 2.6 Data synthesis and statistical analysis

EndNote X9 was used for literature management, and data were recorded using Excel. Statistical analyses were conducted using RevMan 5.4 and Stata 16.0. Categorical variables were expressed as relative risk (RR) with 95% confidence intervals (CIs). Continuous variables were expressed as mean difference (MD) or standardized mean difference (SMD) with 95% CIs. The Cochrane Q test and the I^2^ statistic were used to assess heterogeneity. A random-effects model was applied for all analyses when significant heterogeneity was observed (I^2^ ≥ 50% or P < 0.1). This meta-analysis was conducted using a significance level of α = 0.05. The stability of the results and sources of heterogeneity were investigated by subgroup analyses and sensitivity analyses using a leave-one-out approach. Subgroup analysis was performed mainly based on the pregnant women’s age, the number of samples in the included studies, the route of administration, and the postoperative follow-up time. Publication bias was assessed using the funnel plots and Egger’s test. P < 0.05 indicated significant publication bias. Additionally, the quality of evidence for each outcome was assessed using the GRADE approach and classified as high, moderate, low, or very low (Guyatt et al., 2011).

## 3 Results

### 3.1 Study selection

**FIGURE 1:**
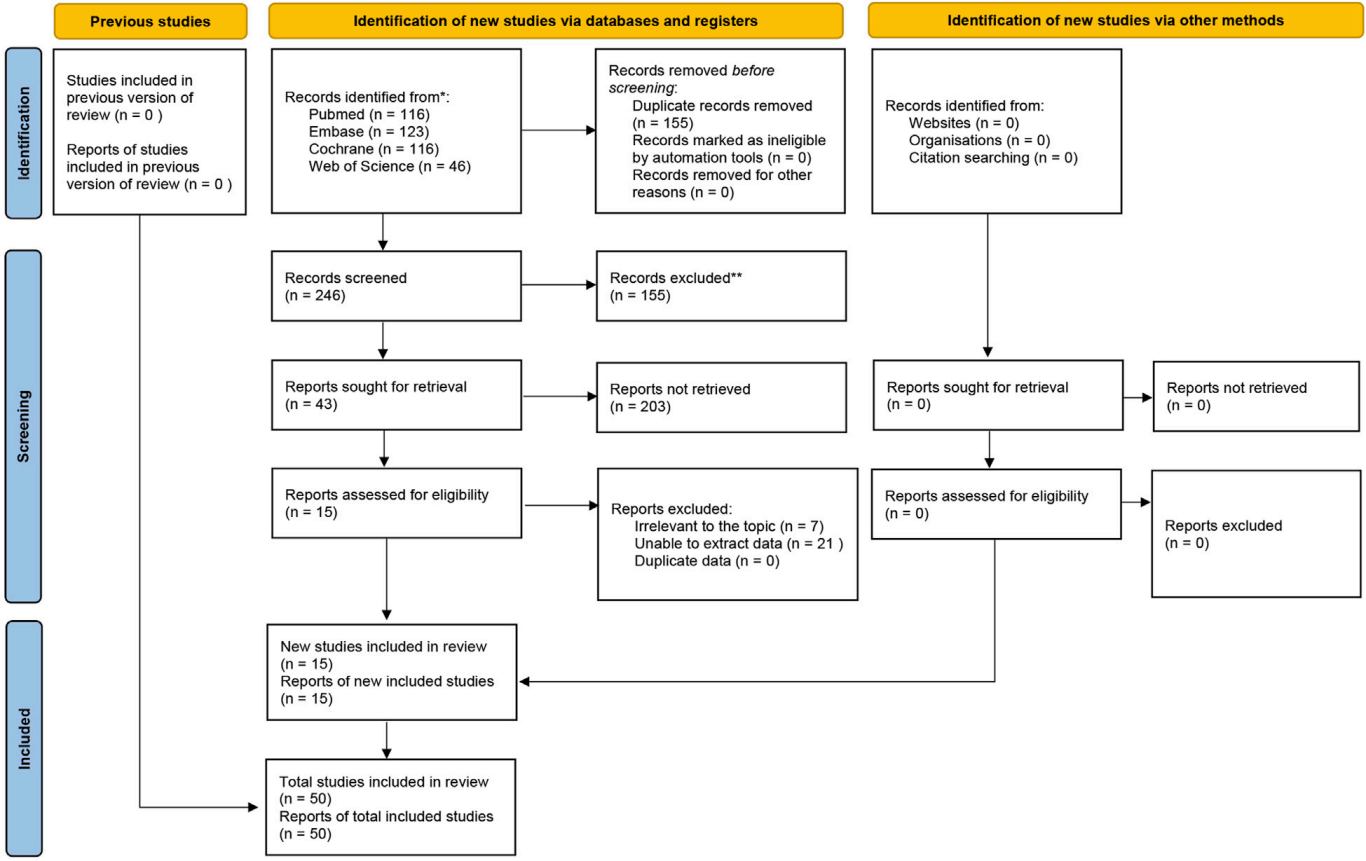
Study screening flow chart.

A total of 401 RCTs were initially identified from the Cochrane Library, Embase, Web of Science, and PubMed. After removing 155 duplicates, 246 studies remained. 203 studies were further excluded after screening the titles and abstracts. The full texts of 43 studies were then reviewed. Ultimately, 10 studies (comprising 15 intervention groups) were included in the analysis. The study selection process is illustrated in Figure 1.

### 3.2 Characteristics of included patients

**FIGURE 2:**
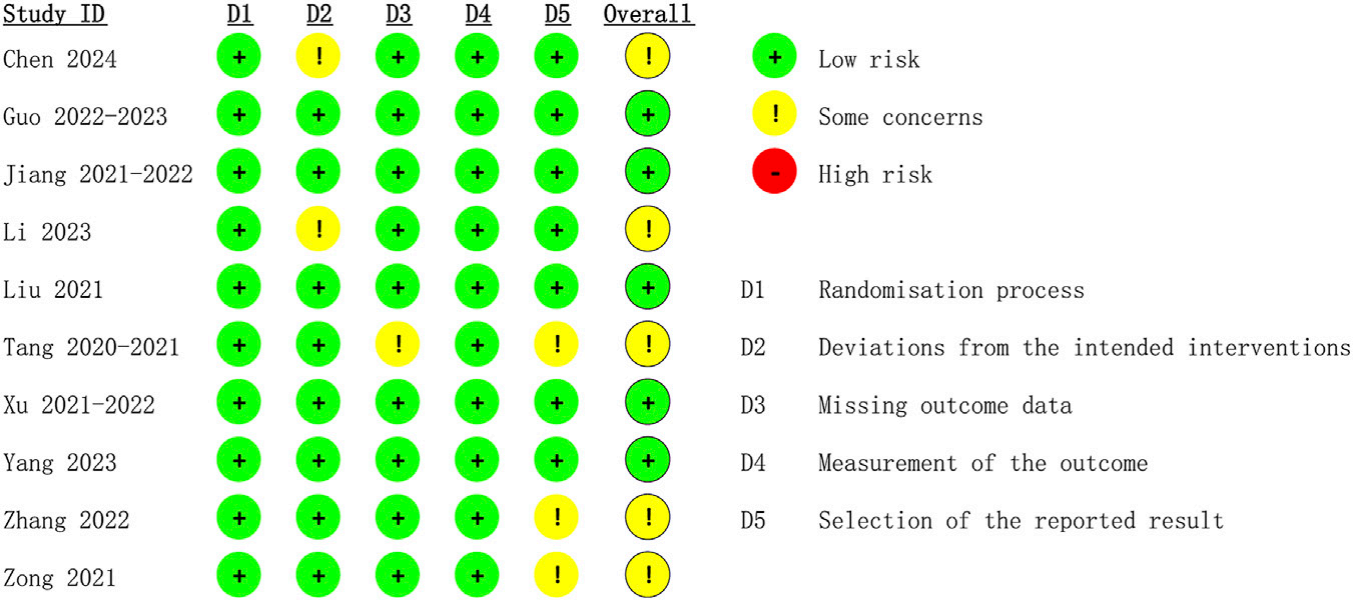
The bias risk assessment results of the included studies.

The 10 eligible studies comprising 15 arms were all from China. A total of 2218 patients were included in this meta-analysis. The average age of patients ranged from 26.66 ± 3.69 to 33.35 ± 5.29 years, and BMI values ranged from 24.1 ± 3.1 to 29 ± 7.39. The ASA classifications of patients ranged from I to II. Table 1 illustrates the characteristics of the included studies. The 15 arms all had a low risk of bias in reporting and random sequence generation. Two arms had an unclear risk of selection bias. Three arms had an unclear risk of bias in outcome assessment. Three arms had an unclear risk of bias in data completeness. Two arms had an unclear risk of other bias (as illustrated in Figure 2).

**TABLE 1:** The basic information of patients.

Study design	Registration number	Population				Intervention	Control	Route of administration
		Gestational hypertension	Gestational diabetes	Gestationalhypothyroidism	Scarred uterus			
RCT		Unclear	Unclear	Unclear	Intervention group: 26/75	After delivering the fetus intraoperatively: ES 0.15 mg/kg IV	After delivering the fetus intraoperatively: Equivalent NS IV	Intravenous injection
					Control group: 25/75	Postoperation: suf100ug + ES1.25 mg/kg IV	Postoperation:suf 100ug IV	Intravenous injection
RCT	ChiCTR2200067054	Intervention group: 21/124	Intervention group: 12/124	Intervention group: 35/124	Unclear	Postoperation:suf 2ug/kg + es 1.5 mg/kg IV	Postoperation:suf 2ug/kg IV	Intravenous injection
		Control group:24/122	Control group: 13/122	Control group:32/122				
RCT	ChiCTR2100046049	Intervention group: 9/62	Intervention group:9/62	Unclear	Intervention group: 39/62	Anesthesia method: 9–11 mg 0.5%bup SA	Anesthesia method:9–11 mg 0.5%bup SA	Subarachnoid drug administration
		Control group: 7/61	Control group: 8/61		Control group:40/61	After delivering the fetus:ES 10 mg IVD	After delivering the fetus: Equivalent NS	Intravenous pump injection
						Postoperation:PCIA:SUF 100 μg + ES 1.25 mg/kg	Postoperation: PCIA:suf 100ug	Intravenous pump injection
RCT		Unclear	Unclear	Unclear	Unclear	After delivering the fetus: ES 0.25 mg/kg· IV	After delivering the fetus: Equivalent saline solution	Intravenous injection
RCT		Unclear	Unclear	Unclear	Unclear	Preincision: 0.25 mg/kg ES	Equivalent placebo	Epidural injection
RCT	NCT05582135	Unclear	Unclear	Unclear	Unclear	ES 0.25 mg/kg	Morphine 2 mg	Epidural injection
RCT	NCT05582137	Unclear	Unclear	Unclear	Unclear	ES 0.25 mg/kg+2 mg morphine	Morphine 2 mg	Epidural injection
RCT	NCT05582136	Unclear	Unclear	Unclear	Unclear	ES 0.25 mg/kg+1 mg morphine	Morphine 2 mg	Epidural injection
RCT	NCT05414006	E1:1	E1:6	E1:4	E1:12	After delivering the fetus: ES 0.1 mg/Kg EP + PCEA	Equivalent saline solution + PCEA	Epidural injection +pump injection
		C1:2	C1:7	C1:1	C1:13			
RCT	NCT05414006	E2:2	E2:9	E2:3	E2:10	After delivering the fetus: ES 0.1 mg/kg IV + PCIA	Equivalent saline solution + PCIA	Intravenous injection + Intravenous pump injection
		C2:2	C2:6	C2:6	C2:7			
RCT		Unclear	Unclear	Unclear	Unclear	After delivering the fetus: ES 0.15 mg/kg EP	10 ML NS	Epidural injection
RCT		Unclear	Unclear	Unclear	Unclear	After delivering the fetus: ES 0.25 mg/kg EP	10 ML NS	Epidural injection
RCT	ChiCTR2300071350	Unclear	Unclear	Unclear	Unclear	After delivering the fetus: 10min 0.3 mg/kg ES + 0.5ug/kg dextromethorphan IV	After delivering the fetus: 0.5ug/kg dextromethorphan IV	Intravenous injection
RCT	ChiCTR2300071350	Unclear	Unclear	Unclear	Unclear	After delivering the fetus: 10min 0.3 mg/kg ES + 0.5ug/kg dextromethorphan IV	After delivering the fetus: Equivalent saline solution IV	Intravenous injection
RCT	ChiCTR2200062848	Unclear	Unclear	Unclear	Unclear	PCIA: es1mg/kg + Tramadol 400 mg	PCIA: Butorphanol 0.1 mg/kg + Tramadol 400 mg	Intravenous pump injection

Time of administration	Patients		Patients	Mean follow-up/1W	Mean/median age/30Y		Mean/median BMI	
	Intervention group	Control group	Total number of participants/150 limit		Intervention group	Control group	Intervention group	Control group
After the baby is delivered	59	62	121	6M	30.2 ± 4.1	30.4 ± 4.3		
After the operation								
After the operation	124	122	246	42D	28 ± 3.1	27.7 ± 4.8	24.5 ± 4.7	24.1 ± 3.1
Before the operation begins	62	61	123	6M	30.3 ± 4.1	29.8 ± 4.2	28.4 ± 3.7	28.7 ± 3.8
Continue to use after the delivery of the fetus								
Continue to use after the operation								
After the baby is delivered	70	70	140	6W	25.41 ± 2.77	25.87 ± 2.65	26.11 ± 3.08	25.99 ± 3.14
Before the operation begins	300	300	600		30.4 (4.2)	30.9 (4.3)	25.9 (1.9)	25.9 (2.2)
After the operation	30	30	60	2D	27.83 ± 4.11	27.67 ± 3.63	27.70 ± 2.70	27.01 ± 2.55
After the operation	30	30	60	2D	28.20 ± 4.06	27.67 ± 3.63	26.78 ± 2.70	27.01 ± 2.55
After the operation	29	30	59	2D	26.66 ± 3.69	27.67 ± 3.63	28.23 ± 2.29	27.01 ± 2.55
Continue to use after the delivery of the fetus	29	30	59	24H	31.2 ± 3.4	32 ± 4.1	27.0 ± 3.3	26.9 ± 3.0
Continue to use after the delivery of the fetus	29	30	59	24H	31.4 ± 4.0	30.3 ± 4.3	27.5 ± 3.7	26.7 ± 3.2
After the baby is delivered	97	98	195	42D	32 ± 4	33 ± 4	28.8 ± 3.3	29.0 ± 2.8
	97	98	195	42D	33 ± 8	33 ± 4	28.9 ± 2.9	29.0 ± 2.8
After the baby is delivered	71	73	144	1D	33.35 ± 5.29	31.35 ± 5.29	26.57 ± 28.51	26.87 ± 3.50
	71	74	145	1D	33.35 ± 5.29	32.65 ± 5.29	26.57 ± 2.90	27.28 ± 3.35
Continue to use after the operation	85	85	170	—	28 ± 10.56	30 ± 13.57	29 ± 7.39	28.4 ± 9.05

### 3.3 Postoperative analgesia scores

#### 3.3.1 Rest pain scores at 24 hours postoperatively

**FIGURE 3:**
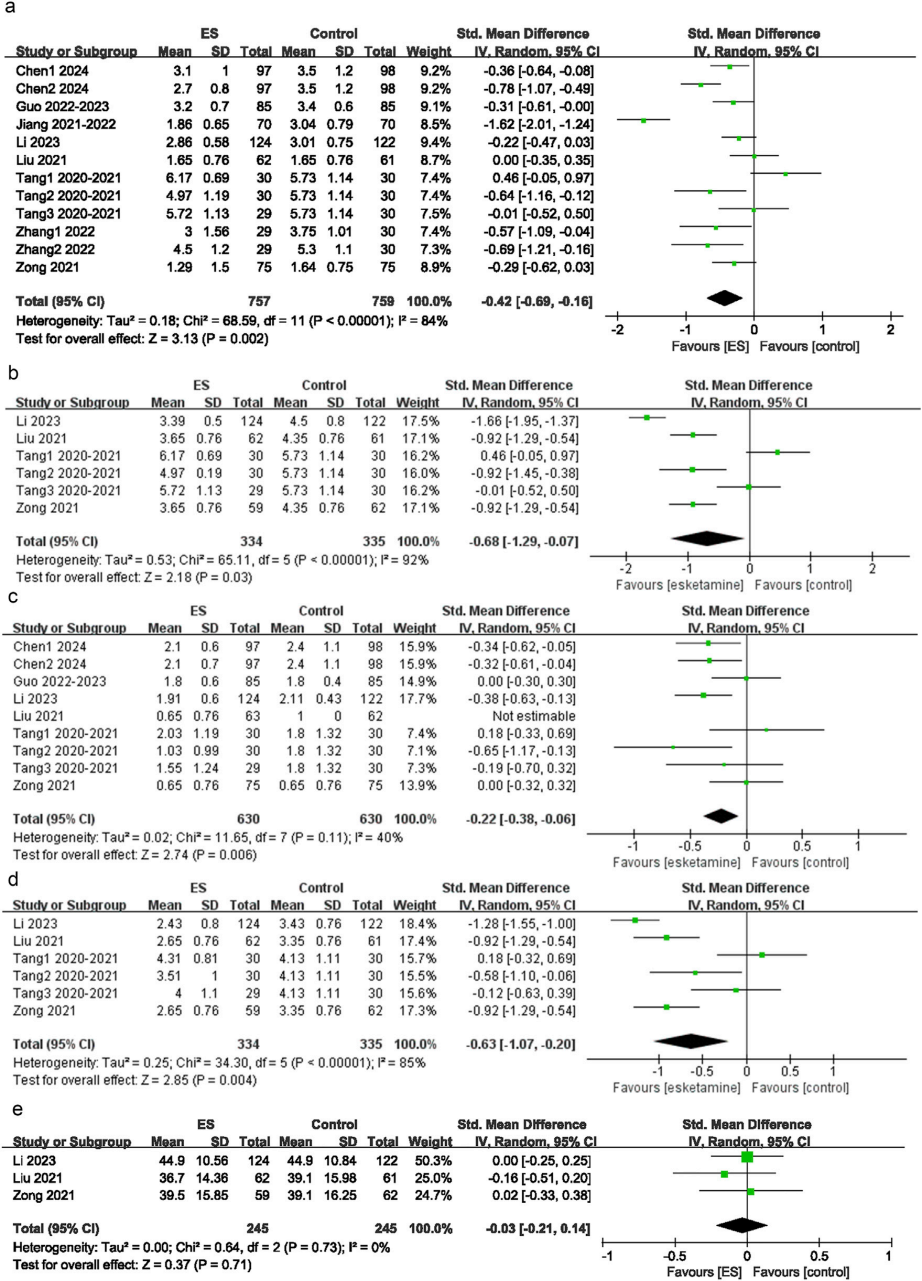
Depression scores. **(a)** Effect of esketamine on rest pain at 24 h after surgery, **(b)** Effect of esketamine on restpain at 48 h after surgery, **(c)** Effect of esketamine on movement-evoked pain at 24 h after surgery, **(d)** Effect of esketamine on movement-evoked pain at 48 h after surgery, **(e)** Effect of esketamine on the consumption of sufentanil.

Among the 15 intervention groups, 12 reported rest pain scores at 24 h postoperatively. The pooled results indicated that, between the control group and the esketamine group, a significant difference was observed in rest pain scores at 24 h postoperatively (SMD = −0.42; 95% CI: −0.69 to −0.16; P < 0.00001; I^2^ = 84%) (Figure 3a).

#### 3.3.2 Rest pain scores at 48 hours postoperatively

Among the 15 intervention groups, nine recorded rest pain scores at 48 h postoperatively. The pooled results indicated a significant difference in rest pain scores at 48 h postoperatively between the control group and the esketamine group (SMD = −0.22; 95% CI: 0.38 to −0.06; P = 0.006; I^2^ = 54%) (Figure 3b).

#### 3.3.3 Movement-evoked pain (MEP) scores at 24 hours postoperatively

Among the 15 intervention groups, six recorded pain scores on movement or coughing at 24 h postoperatively. The pooled results suggested that, compared with the control group, MEP scores at 24 h postoperatively were significantly lower in the esketamine group (SMD = −0.68; 95% CI: −1.29 to −0.07; P < 0.00001; I^2^ = 92%) (Figure 3c). This significant difference (P < 0.05) indicated that esketamine may effectively alleviate pain triggered by movement or coughing within 24 h postoperatively.

#### 3.3.4 MEP scores at 48 hours postoperatively

Among the 15 intervention groups, six reported pain scores on movement or coughing at 48 h postoperatively. The pooled results revealed that, compared with the control group, MEP scores at 48 h postoperatively were significantly lower in the esketamine group (SMD = −0.63; 95% CI: −1.07 to −0.20; P < 0.00001; I^2^ = 85%) (Figure 3d). This significant difference (P < 0.05) suggested that esketamine may effectively alleviate pain triggered by movement or coughing within 48 h postoperatively.

#### 3.3.5 Sufentanil consumption

Among the 15 included studies, three reported changes in sufentanil consumption. The pooled results indicated that, compared with the control group, esketamine did not significantly reduce the consumption of sufentanil (SMD = −0.03; 95% CI: −0.21 to 0.14; P = 0.71; I^2^ = 0) (Figure 3e). This result suggested that esketamine may not effectively decrease the consumption of sufentanil.

### 3.4 EPDS scores

**FIGURE 4:**
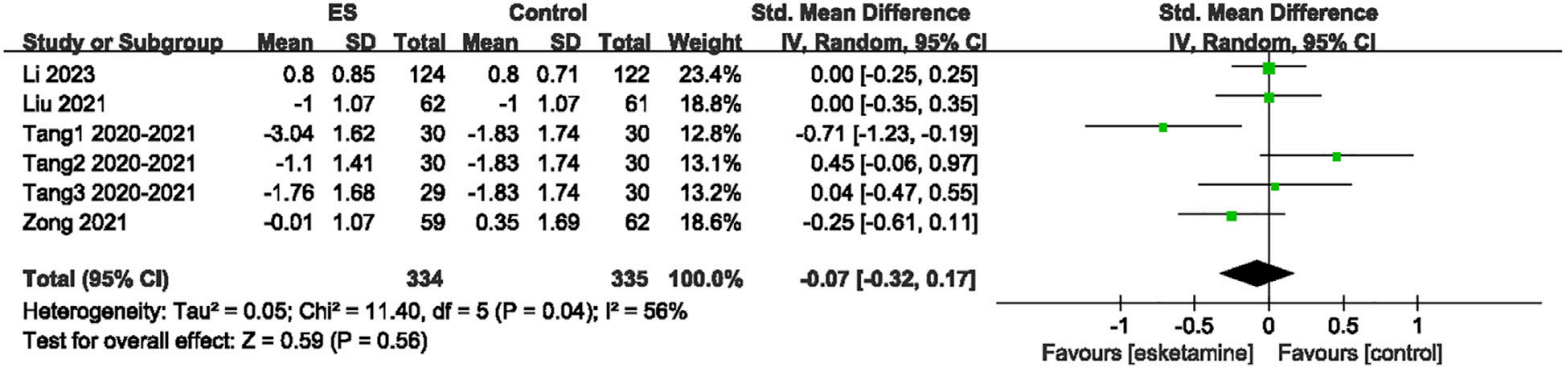
Effect of esketamine on EPDS score.

Out of the 15 intervention groups, five evaluated the EPDS scores before and after surgery. Compared to the control group, the EPDS scores were significantly reduced in the esketamine group (SMD = −0.21; 95% CI: −0.39 to −0.04; P = 0.02; I^2^ = 45%) (Figure 4). This result suggested that esketamine may effectively alleviate postpartum depression in patients undergoing CD.

### 3.5 Postoperative adverse events

#### 3.5.1 Nausea and vomiting

**FIGURE 5:**
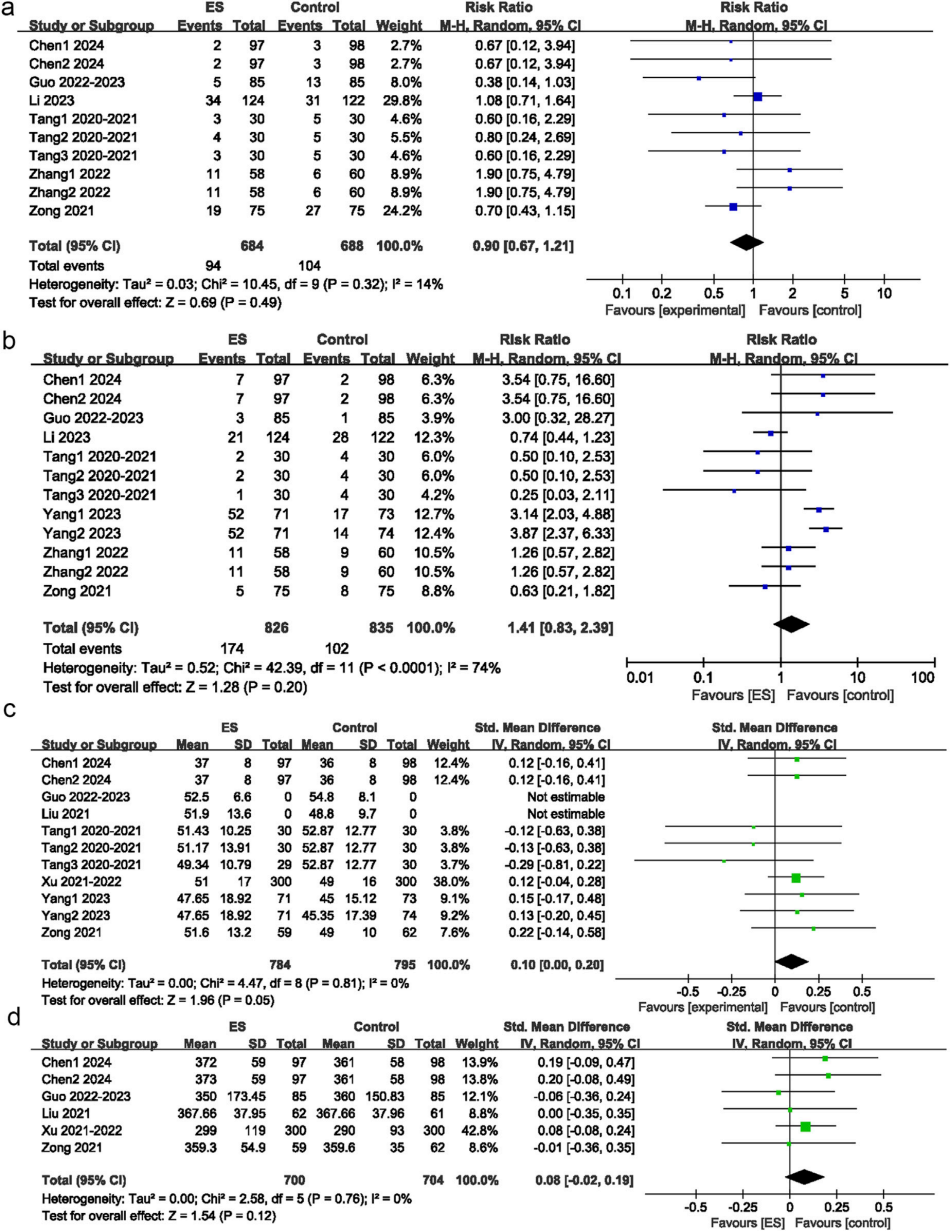
Postoperative adverse reaction. **(a)** Effect of esketamine on postoperative nausea and vomiting, **(b)** Effect of esketamine on dizziness, **(c)** Effect of esketamine on operation duration, **(d)** Effect of esketamine on intraoperative blood loss.

Among the 15 intervention groups, 10 recorded the incidence of PONV. No significant difference in the incidence of PONV was observed between the esketamine and control groups (RR = 0.90; 95% CI: 0.67 to 1.21; P = 0.49; I^2^ = 14%) (Figure 5a).

#### 3.5.2 Dizziness

Among 15 intervention groups, 12 recorded the incidence of postoperative dizziness. No significant difference in the incidence of postoperative dizziness was observed between the esketamine and control groups (RR = 1.41; 95% CI: 0.83 to 2.39; P = 0.2; I^2^ = 74%) (Figure 5b)

### 3.6 Operation duration

Among 15 intervention groups, 11 reported operation duration. No significant difference in operation duration was observed between the esketamine and control groups (SMD = 0.07; 95% CI: −0.04 to 0.17; P = 0.22; I^2^ = 16%) (Figure 5c).

### 3.7 Intraoperative blood loss

Among 15 intervention groups, six reported intraoperative blood loss. No significant difference in intraoperative blood loss was observed between the esketamine and control groups (SMD = 0.08; 95% CI: -0.02 to 0.19; P = 0.12; I^2^ = 0%) (Figure 5d).

### 3.8 Sensitivity analysis

**FIGURE 6:**
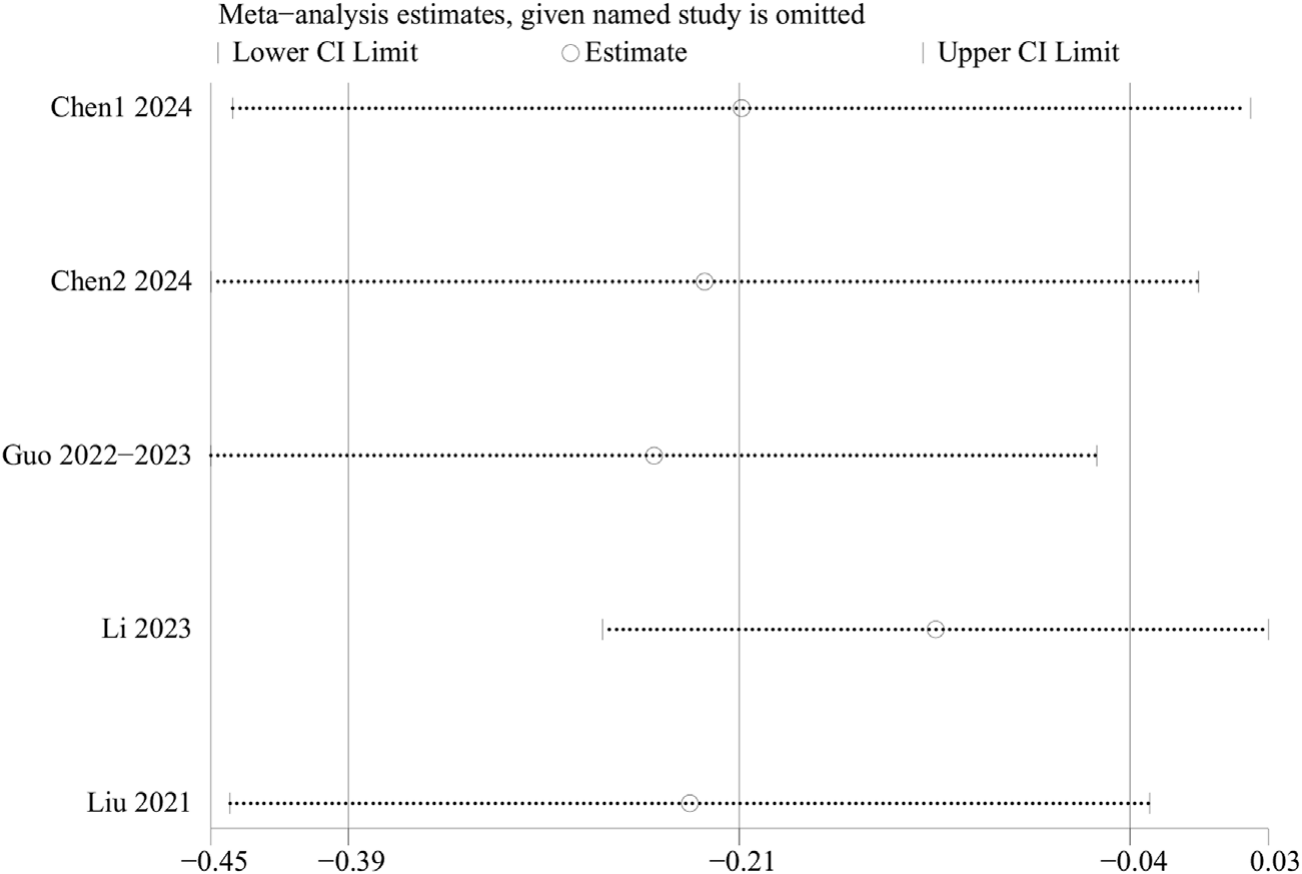
Sensitivity analysis of EPDS.

Sensitivity analyses for EPDS scores, intraoperative blood loss, operation duration, rest pain, MEP, overall adverse events, PONV, and postoperative dizziness were conducted using a leave-one-out approach. The results demonstrated that, except for the EPDS scores, the results for other outcome indicators were stable and not significantly affected by the exclusion of any single study. However, sensitivity analysis for the EPDS scores indicated significantly unstable results. Thus, the above results should be interpreted with caution. In terms of EPDS scores, when data from the RCTs by Li et al. or Chen et al. were excluded, heterogeneity disappeared, suggesting that these two studies contributed substantially to the observed heterogeneity (Figure 6). Because the results of EPDS scores were unstable, the results of EPDS in this study were only exploratory.

### 3.9 Publication bias assessment

**FIGURE 7:**
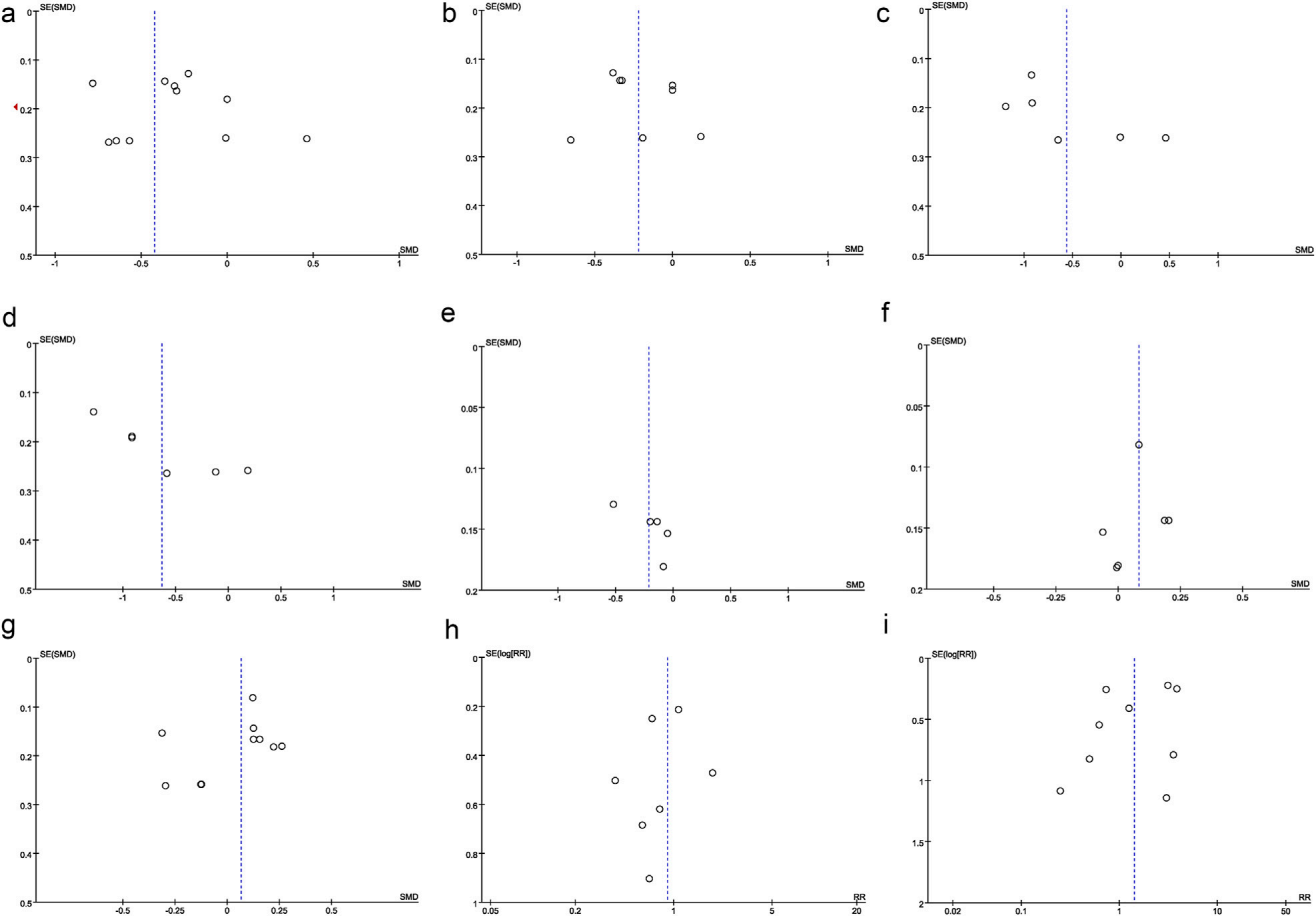
Other perioperative indicators. **(a)** Funnel plot of operation time, **(b)** Funnel plot of postoperative rest pain, **(c)** Funnel plot of postoperative MEP pain, **(d)** Funnel plot of postoperative overall pain, **(e)** Funnel plot of EPDS, **(f)** Funnel plot of intraoperative blood loss, **(g)** Funnel plot of operation duration, **(h)** Funnel plot of PONV, **(i)** Funnel plot of postoperative dizziness.

Egger’s test was performed to evaluate potential publication bias for the following outcomes: rest pain at 24 h, MEP at 24 h, rest pain at 48 h, MEP at 48 h, EPDS scores, intraoperative blood loss, operation duration, PONV, and postoperative dizziness. The results indicated no significant publication bias (P > 0.05) for any of these outcomes. Additionally, funnel plots were generated for rest pain at 24 h (Figure 7a), MEP at 24 h (Figure 7b), rest pain at 48 h (Figure 7c), MEP at 48 h (Figure 7d), EPDS scores (Figure 7e), intraoperative blood loss (Figure 7f), operation duration (Figure 7g), PONV (Figure 7h), and postoperative dizziness (Figure 7i). The results indicated no significant publication bias.

### 3.10 Subgroup analysis

Subgroup analyses based on the presence or absence of postoperative nausea, dizziness, and operation duration were carried out. Subgroup analyses for primary outcomes were conducted based on the route of administration. For adverse postoperative outcomes, subgroup analyses stratified by sample size, maternal age, and postoperative follow-up duration were performed. Regarding primary outcomes, compared with those in the epidural analgesia group, patients receiving intravenous analgesia experienced significantly lower rest pain at 24 h (SMD = −0.51; 95% CI: −0.94 to −0.08; P = 0.02; I^2^ = 90%). However, for rest pain at 48 h, compared with intravenous administration, epidural analgesia was more effective for relieving pain (SMD = −0.29; 95% CI: −0.49 to −0.08; P = 0.006; I^2^ = 27%). Additionally, subgroup analysis by age revealed that older patients had significantly higher rest pain scores at both 24 h (SMD = −0.43; 95% CI: −0.67 to −0.19; P = 0.0005; I^2^ = 63%) and 48 h postoperatively (SMD = −0.23; 95% CI: −0.44 to −0.03; P = 0.03; I^2^ = 32%). The incidence of postoperative dizziness was significantly higher in patients older than 30 years (RR = 2.08; 95% CI: 1.26 to 3.34; P = 0.004; I^2^ = 64%). However, such adverse events were not found in parturients younger than 30 years old (RR = 0.70; 95% CI: 0.45 to 1.09; P = 0.12; I^2^ = 0%). These results suggested that age should be considered when determining whether to use esketamine. No significant difference was found in other subgroup analyses. The detailed subgroup analyses are illustrated in Table 2.

**TABLE 2:** Subgroup analysis.

Subgroup	Nause/Vomit				Dizzy				Operation time			
	Study	RR [95%CI]	*P* Value	*I* ^2^	Study	RR [95%CI]	*P* Value	*I* ^2^	Study	SMD [95%CI]	*P* Value	*I* ^2^
Total	10	0.90 [0.67, 1.21]	0.49	14%	12	1.41 [0.83, 2.39]	0.2	74%	11	0.07 [-0.04, 0.17]	0.22	16%
Sample size												
≥150	4	0.79 [0.46, 1.35]	0.39	22%	4	1.86 [0.64, 5.40]	0.26	61%	5	0.07 [-0.10, 0.23]	0.43	49%
<150	6	0.97 [0.62, 1.51]	0.89	25%	8	1.30 [0.70, 2.39]	0.4	74%	6	0.06 [-0.10, 0.22]	0.47	0%
Mean/median age												
≥30y	5	1.08 [0.63, 1.84]	0.79	34%	7	2.08 [1.26, 3.44]	0.004	64%	8	0.10 [-0.01, 0.21]	0.08	18%
<30y	5	0.82 [0.56, 1.21]	0.33	8%	5	0.70 [0.45, 1.09]	0.12	0%	3	−0.18 [-0.47, 0.11]	0.23	0%
Follow-up												
≥1week	5	0.80 [0.58, 1.12]	0.19	10%	4	1.23 [0.53, 2.85]	0.62	58%	—	—	—	—
<1 week	5	1.19 [0.71, 1.99]	0.52	8%	7	1.48 [0.80, 2.74]	0.22	72%	—	—	—	—

Subgroup	24-h resting pain				48-h resting pain			
	Study	SMD [95%CI]	*P* Value	*I* ^2^	Study	SMD [95%CI]	*P* Value	*I* ^2^
Total	12				9			
Route of administration								
Epidural administration	6	−0.34 [-0.68,0.01]	0.06	76%	5	−0.29 [−0.49,-0.08]	0.006	27%
Intravenous injection	6	−0.51 [-0.94,-0.08]	0.02	90%	4	−0.14 [−0.41,-0.12]	0.3	60%

Subgroup	24-h resting pain				48-h resting pain			
	Study	SMD [95%CI]	*P* Value	*I* ^2^	Study	SMD [95%CI]	*P* Value	*I* ^2^
Total	12				9			
Sample size								
≥150	4	−0.41 [−0.66, −0.17]	0.0008	66%	4	−0.27 [−0.44,-0.11]	0.001	29%
<150	8	−0.42 [−0.88, 0.03]	0.07	88%	5	−0.14 [−0.47,0.18]	0.38	50%
Mean/median age								
≥30y	6	−0.43 [−0.67, −0.19]	0.0005	63%	4	−0.23 [−0.44,-0.03]	0.03	32%
<30y	6	−0.40 [−0.92, 0.12]	0.13	91%	5	−0.21 [−0.47,0.05]	0.11	54%

### 3.11 GRADE assessment

According to the GRADE evaluation, the quality of evidence of rest pain at 24 h, rest pain at 48 h, sufentanil consumption, EPDS scores, operation duration, and intraoperative blood loss was moderate. The quality of evidence for MEP at 24 h, MEP at 48 h, and the incidence of postoperative dizziness was low. No outcomes were rated as high-quality or very low-quality evidence. Detailed GRADE ratings are presented in Table 3.

**TABLE 3:** Grading of recommendations assessment.

No. of studies	Outcomes	RR/SMD	95%CI	*I* ^2^; P value	Risk of bias	Inconsistency	Indirectness	Imprecision	Publication bias	Plausible confounding	Magnitude of Effect	Dose-response gradient	GRADE
12	Rest pain scores at 24 hours	−0.42	−0.69, −0.16	84%; P < 0.00001	No serious risk	Serious inconsistency	No serious indirectness	No serious imprecision	Undetected	Would not	No	No	Moderate
9	Rest pain scores at 48 hours	−0.22	−0.38, −0.06	40%; P = 0.11	No serious risk	No serious	No serious indirectness	Serious imprecision	Undetected	Would not	No	No	Moderate
6	Movement-evoked pain (MEP) scores at 24 hours	−0.68	−1.29, −0.07	92%; P < 0.00001	No serious risk	Serious inconsistency	No serious indirectness	Serious imprecision	Undetected	Would not	No	No	Low
6	Movement-evoked pain (MEP) scores at 48 hours	−0.63	−1.07,-0.2	85%; P < 0.00001	No serious risk	Serious inconsistency	No serious indirectness	Serious imprecision	Undetected	Would not	No	No	Low
3	Sufentanil consumption	−0.03	−0.21,0.14	0%; P = 0.73	No serious risk	No serious	No serious indirectness	Serious imprecision	Undetected	Would not	No	No	Moderate
5	EPDS scores	−0.21	−0.39,-0.04	45%; P = 0.12	No serious risk	No serious	No serious indirectness	Serious imprecision	Undetected	Would not	No	No	Moderate
12	Dizziness	1.41	0.83,2.39	74%; P < 0.0001	No serious risk	Serious inconsistency	No serious indirectness	Serious imprecision	Undetected	Would not	No	No	Low
10	Nausea and vomiting	0.9	0.67,1.21	14%; P = 0.32	No serious risk	No serious	No serious indirectness	Serious imprecision	Undetected	Would not	No	No	Moderate
11	Operation duration	0.07	−0.04,0.17	16%; P = 0.29	No serious risk	No serious	No serious indirectness	Serious imprecision	Undetected	Would not	No	No	Moderate
6	Intraoperative blood loss	0.08	−0.02,0.19	0%; P = 0.76	No serious risk	No serious	No serious indirectness	Serious imprecision	Undetected	Would not	No	No	Low

## 4 Discussion

Ketamine is the only intravenous anesthetic that can control pain. Esketamine, the S (+) enantiomer of ketamine, exhibits more pronounced pharmacological properties due to its distinct molecular structure. Moreover, it offers both sympathomimetic and rapid antidepressant effects and has been commonly used in pediatric and obstetric anesthesia. During the perinatal period, esketamine, owing to its unique pharmacokinetic properties, takes effect within approximately 30–60 s and lasts approximately 15–20 min. In addition, it exerts relatively mild inhibitory effects on the respiratory and circulatory systems. Therefore, it is particularly suitable for short procedures that require the preservation of spontaneous breathing. For parturients, doses between 0.5 and 1 mg/kg effectively relieve pain without significantly increasing the risk of neonatal abstinence syndrome. Thus, this approach has been incorporated into CD anesthesia guidelines in several countries. In pediatric anesthesia, esketamine notably lowers the incidence of agitation and respiratory complications after surgery due to its good sedative and analgesic effects and its protective role in the respiratory system.

In this study, esketamine demonstrated a significant analgesic effect (Cui et al., 2024; Duan et al., 2024; Bornemann-Cimenti et al., 2016; Autry et al., 2011), supporting its use to control both intraoperative and postoperative pain. Moreover, esketamine can significantly mitigate postoperative depressive symptoms. Subgroup analyses revealed that patients older than 30 years had a higher incidence of dizziness after receiving esketamine. Prior studies have reported that esketamine can reduce morphine and the need for other opioids (Liu et al., 2023). Moreover, when administered at low doses, esketamine can mitigate hyperalgesia (Wang et al., 2021). This study revealed that esketamine had a relatively favorable effect for controlling pain in CD, but overall, the difference was not statistically significant. However, this finding differs from a previous meta-analysis including 11 studies (n = 1855) (Page, 2020), which reports a stronger analgesic effect of esketamine. This difference may be partly due to the smaller sample size, lower quality of evidence, and a lack of subgroup analyses in the previous meta-analysis. In contrast, our study examined adverse effects across subgroups. Furthermore, our results revealed that esketamine could alleviate postoperative depression (Cui et al., 2024; Xu et al., 2023; Suppa et al., 2012; Jiang et al., 2024). This result is consistent with findings reported by prior meta-analyses (Page, 2020; Cumpston et al., 2022).

Our subgroup analyses indicated that older parturients experienced more severe pain, which may be related to the decline in endogenous pain modulation with aging (Hackett et al., 2020).

In addition, our subgroup analyses revealed that esketamine did not increase the incidence of adverse events such as nausea and vomiting in parturients. However, for pregnant women aged over 30 years, the risk of postoperative dizziness may be higher. This may be due to several factors. First, aging can affect the binding function of the GluN2B subunit of NMDA (N-methyl-D-aspartate) receptors (Pegasiou et al., 2020). Due to the physiological changes in parturients, biomarkers such as the levels of thyroid hormone are not rapidly adjusted before surgery (Geno and Nerenz, 2022). As they age, the level of gonadotropins (LH and FSH) rises, while the production of ovarian hormones (estrogen and progesterone) decreases (van den Beld et al., 2018). These hormonal fluctuations between the preoperative and postoperative periods may lead to an increased risk of postoperative dizziness. Additionally, even within the same individual, the metabolism of antidepressants can differ significantly between the pregnant and non-pregnant states (Schoretsanitis et al., 2020; Pinheiro and Stika, 2020). Moreover, pregnant women often have heightened alertness and greater mental stress compared to non-pregnant women. Prolonged stress may increase the incidence of adverse events (Michopoulos et al., 2015). Second, some patients may experience supine hypotensive syndrome during surgery, thereby increasing the risk of postoperative dizziness (Ikeda et al., 1992). Compared to other surgeries, CD can cause more intense pain and higher intraoperative stress levels. CD with significant blood loss may even cause stroke (Miller and Leffert, 2020). Third, in some of the included RCTs, esketamine is administered combined with opioids (Liu et al., 2023; Li et al., 2024; Qiankun et al., 2023), and opioid use can increase the incidence of postoperative dizziness (Ikeda et al., 1992). Previous meta-analyses have demonstrated that prolonged use of opioids after surgery increases the incidence of adverse events (Wang et al., 2022). Therefore, the individualized and precise administration of this drug is required (Parsaei et al., 2024). Given comparable effects, anesthesia without opioids may be more conducive to reducing the incidence of postoperative adverse events (Johnston and Zarate, 2024). Although mild to moderate adverse reactions (such as dizziness and nausea) are observed, strict adherence to risk stratification and monitoring procedures (such as avoiding driving and contraindications to concurrent medication) is essential in clinical practice (Di Vincenzo et al., 2024). This is consistent with the monitoring indicators within 24 h after surgery included in this study, but long-term safety still needs to be verified with a larger sample size (Di Vincenzo et al., 2024).

Esketamine, a relatively novel analgesic agent, exerts its effects by modulating the N-methyl-D-aspartate (NMDA) receptor. Compared with traditional ketamine, esketamine shows no significant difference in mitigating depression (Geno and Nerenz, 2022). In clinical practice, esketamine has demonstrated effective analgesic effects when administered via intravenous injection, intramuscular injection, or even nasal spray. It also shows notable analgesic effects when used in combination with general anesthesia through epidural injection. (Schoretsanitis et al., 2020). Our study demonstrated that esketamine had potent analgesic effects, although its impact on controlling rest pain was less significant than on MEP. Several factors may explain this finding. Firstly, esketamine primarily exerts its analgesic effects by antagonizing the N-methyl-D-aspartate receptor, but it also interacts with opioid receptors. Compared with opioids for a single target, esketamine targets multiple receptors, which may enhance the efficacy for relieving pain and reduce the patient’s sensitivity to pain stimuli. An earlier study has reported that esketamine does not have significant analgesic effects, but esketamine at a low dose can indeed attenuate pain hypersensitivity (Pinheiro and Stika, 2020). Secondly, all the studies included in our analysis employed neuraxial anesthesia. Properly administered neuraxial anesthesia can significantly reduce the need for painkillers and lower pain scores (Subramanian et al., 2022). Preclinical studies suggest that esketamine may be particularly effective for controlling complex chronic pain and neuropathic pain (Michopoulos et al., 2015; Ikeda et al., 1992). This may explain why its analgesic effect of rest pain was less pronounced than that in MEP in our study. The results of MEP in this study were obtained using the DerSimonian-Laird method, without Hartung-Knapp modification. Therefore, the possibility of false positives cannot be ruled out, meaning that the effect of the drug on exercise-induced pain may not be as significant as expected, and a larger sample analysis may be needed. Lastly, variations in administration routes and dosages across the included studies could contribute to differences in analgesic effects. Thus, further meta-analyses comparing different administration routes are required, and larger-scale studies with standardized drug regimens are needed to fully establish the analgesic efficacy of esketamine.

Depression and pain, which are closely interrelated, can mutually influence each other, and they coexist within neural circuits (Subramanian et al., 2022). Two RCTs (Li et al., 2024; Tang et al., 2022) have demonstrated that esketamine relieves postoperative pain on days 1 and 2. Whether administered intravenously or via intrathecal injection, esketamine effectively reduces NRS pain scores at 2, 4, 8, 12, 24, and 48 h after CD. In the study by Li et al., a significant difference is observed in EPDS scores before and after surgery. Their results suggest that effective postoperative analgesia can substantially reduce postpartum depression scores and improve postoperative recovery. While a decrease in the total EPDS score reflects symptom relief, patient-reported outcomes (such as loss of suicidal ideation, anhedonia) may be more sensitive in capturing the recovery of emotional function (Di Nicola et al., 2025). This suggests that the depression status of patients needs to be assessed using multidimensional assessment tools (Di Nicola et al., 2025). One study (Williams et al., 2018) has demonstrated that when patients receive both an opioid receptor antagonist and esketamine, a statistical difference in postpartum depression scores is found between the experimental and control groups. Their results indicate a potential relationship between the antidepressant effects of esketamine and the receptor activity of opioids. Similar findings are also reported by other preliminary studies (Jiang et al., 2024).

In addition to pain control, esketamine can provide antidepressant effects by interacting with NMDA receptors. NMDA receptors comprise multiple subunits, such as GluN2A and GluN2B. Both esketamine and its metabolites can interact with these subunits to produce antidepressant effects (Bornemann-Cimenti et al., 2016). Brain-derived neurotrophic factor (BDNF) is important in mediating the antidepressant properties of esketamine by modulating synaptic plasticity. In a preclinical study, mice lacking BDNF exhibit a significantly reduced sensitivity to ketamine (Autry et al., 2011). Thus, an increase in the level of blood BDNF may help lower the risk of developing postpartum depression, which aligns with the findings reported by Liu et al. (2023). Intravenous administration of esketamine during surgery can elevate postoperative BDNF levels in parturients, thereby alleviating postoperative depression.

This study has several limitations. Firstly, as 2218 patients were all from China, the generalizability of the findings is limited. Secondly, our meta-analysis primarily focuses on pain and depression, whereas neonatal outcomes, such as Apgar scores and cord blood pH, are not evaluated. Thirdly, the included studies predominantly report short-term outcomes (within 7 days), and patients from long-term follow-up are scarce. Additionally, the selected RCTs covered five routes of administration (including single intravenous injection, continuous intravenous infusion, single intravenous injection plus continuous intravenous infusion, single intramuscular injection plus continuous intravenous infusion, and single intravertebral injection) and four different administration times, including intraoperative (pre-incision and post-delivery interventions), postoperative, and intraoperative plus postoperative. Moreover, dosages varied widely from 0.1 mg/kg to 0.3 mg/kg. As a result, subgroup analyses based on a specific route of administration and dosage are not carried out. Lastly, since depression is only evaluated by EPDS, the results may be influenced by patients’ subjective perceptions. Individual differences in the improvement of EPDS score suggest that the efficacy may be modulated by baseline psychological characteristics (such as cognitive flexibility) or sensitivity to assessment tools (Olivola et al., 2025). Although this study focuses on the perinatal period, further exploration of biomarkers for risk stratification of patients is needed, rather than overemphasizing the role of a single drug (Olivola et al., 2025).

## 5 Conclusion

Esketamine demonstrates favorable efficacy in pain control and may reduce the risk of postpartum depression in patients undergoing CD. Subgroup analyses indicate that younger parturients receiving esketamine had a lower risk of developing postoperative adverse events. Considering the limitations of our study, such as potential heterogeneity and relatively small sample size, future multicenter, large-scale prospective RCTs are required to validate the efficacy of esketamine in improving the recovery of patients undergoing CD.

Regarding the antidepressant effect of esketamine, some heterogeneity is observed. Therefore, the results need to be further validated and should be interpreted with caution. Moreover, since the drug is currently approved and used only in China, all trial participants were Chinese. Furthermore, one of the included studies is an English translation of a Chinese article (Miller and Leffert, 2020). Thus, the findings may only be applicable to the Chinese population. Multicenter studies involving diverse ethnic groups in other countries are encouraged to further evaluate the efficacy and safety of esketamine.

## Data Availability

The original contributions presented in the study are included in the article/Supplementary Material, further inquiries can be directed to the corresponding author.
