# Supplementary material for: Efficacy and safety of esketamine for pain control after cesarean delivery: a systematic review and meta-analysis of randomized controlled trials
Source: Front Pharmacol. 2025 Dec 1;16:1708221. doi: 10.3389/fphar.2025.1708221 (PMC12702921; doi:10.3389/fphar.2025.1708221)
Supplement: Supplementary file 1 [file Table1.docx]

Table S1 illustrates the search strategy

Pubmed-57

(((((((“Esketamine” [Supplementary Concept]) OR ((-)-Ketamine)) OR (S-Ketamine)) OR (Kataved)) OR (Spravato)) AND ((((((((((((“Cesarean Section”[Mesh]) OR (Cesarean Sections)) OR (Abdominal Delivery)) OR (C-Section (OB))) OR (C Section (OB))) OR (C-Sections (OB))) OR (Caesarean Section)) OR (Caesarean Sections)) OR (Delivery, Abdominal)) OR (Abdominal Deliveries)) OR (Deliveries, Abdominal)) OR (Postcesarean Section))) AND (((((((((((((((((((((((((((“pain”[Mesh]) OR (Suffering, Physical)) OR (Physical Suffering)) OR (Physical Sufferings)) OR (Sufferings, Physical)) OR (Ache)) OR (Aches)) OR (Pain, Burning)) OR (Burning Pain)) OR (Burning Pains)) OR (Pains, Burning)) OR (Pain, Crushing)) OR (Crushing Pain)) OR (Crushing Pains)) OR (Pains, Crushing)) OR (Pain, Migratory)) OR (Migratory Pain)) OR (Migratory Pains)) OR (Pains, Migratory)) OR (Pain, Radiating)) OR (Pains, Radiating)) OR (Radiating Pain)) OR (Radiating Pains)) OR (Pain, Splitting)) OR (Pains, Splitting)) OR (Splitting Pain)) OR (Splitting Pains))) AND (random*)

Embase-123/


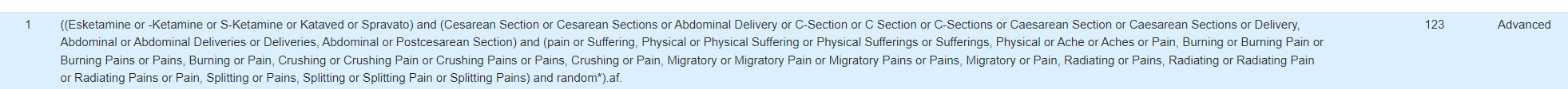


Cochrane-116


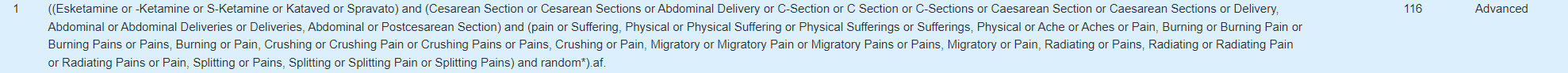


Web of science-47

**(((((((Esketamine) OR (-Ketamine)) OR (S-Ketamine)) OR (Kataved)) OR (Spravato)) AND ((((((((((((Cesarean Section) OR (Cesarean Sections)) OR (Abdominal Delivery)) OR (C-Section)) OR (C Section)) OR (C-Sections)) OR (Caesarean Section)) OR (Caesarean Sections)) OR (Delivery, Abdominal)) OR (Abdominal Deliveries)) OR (Deliveries, Abdominal)) OR (Postcesarean Section))) AND (((((((((((((((((((((((((((pain) OR (Suffering, Physical)) OR (Physical Suffering)) OR (Physical Sufferings)) OR (Sufferings, Physical)) OR (Ache)) OR (Aches)) OR (Pain, Burning)) OR (Burning Pain)) OR (Burning Pains)) OR (Pains, Burning)) OR (Pain, Crushing)) OR (Crushing Pain)) OR (Crushing Pains)) OR (Pains, Crushing)) OR (Pain, Migratory)) OR (Migratory Pain)) OR (Migratory Pains)) OR (Pains, Migratory)) OR (Pain, Radiating)) OR (Pains, Radiating)) OR (Radiating Pain)) OR (Radiating Pains)) OR (Pain, Splitting)) OR (Pains, Splitting)) OR (Splitting Pain)) OR (Splitting Pains))) AND (random*)** (Topic)
